# A review of the tolerability and safety of levocabastine eye drops and nasal spray. Implications for patient management

**DOI:** 10.1155/S0962935195000810

**Published:** 1995-05

**Authors:** Peter H. Howarth

**Affiliations:** 1 University of Medicine Level D, Centre Block Southampton General Hospital Tremona Road Southampton S09 4XY UK

## Abstract

Levocabastine is a highly potent and selective H_1_-receptor antagonist specifically developed for topical administration by ocular and nasal routes. The clinical effects of levocabastine occur rapidly and are predominantly due to local antihistaminic effects at the site of application. Clinically, levocabastine is well tolerated with an adverse effect profile comparable with that of sodium cromoglycate and placebo. As might be expected from the route of drug administration, local irritation is the most frequent adverse event seen with levocabastine eye drops and nasal spray with an incidence comparable with that in placebo-treated controls. Intranasal application of levocabastine has been shown to have no adverse effect on ciliary activity both *in vitro* and *in vivo*, while ocular administration has not been shown to have any significant or consistent adverse effect in both animal and human studies. At therapeutic doses, levocabastine appears to be devoid of significant systemic activity producing no apparent effects on cardiovascular, psychomotor and cognitive function. Since levocabastine undergoes little hepatic metabolism, and only low plasma levels of the drug are attained following topical administration, drug interactions are unlikely.


Mediators of Inflammation 4, S26–S30 (1995)

### A review of the tolerability and safety of levocabastine eye drops and nasal spray. Implications for patient management

Peter H. Howarth

University of Medicine, Level D, Centre Block, Southampton General Hospital, Tremona Road, Southampton SO9 4XY, UK

**Levocabastine is a highly potent and selective $H_1$-receptor antagonist specifically developed for topical administration by ocular and nasal routes. The clinical effects of levocabastine occur rapidly and are predominantly due to local antihistaminic effects at the site of application. Clinically, levocabastine is well tolerated with an adverse effect profile comparable with that of sodium cromoglycate and placebo. As might be expected from the route of drug administration, local irritation is the most frequent adverse event seen with levocabastine eye drops and nasal spray with an incidence comparable with that in placebo-treated controls. Intranasal application of levocabastine has been shown to have no adverse effect on ciliary activity both *in vitro* and *in vivo*, while ocular administration has not been shown to have any significant or consistent adverse effect in both animal and human studies. At therapeutic doses, levocabastine appears to be devoid of significant systemic activity producing no apparent effects on cardiovascular, psychomotor and cognitive function. Since levocabastine undergoes little hepatic metabolism, and only low plasma levels of the drug are attained following topical administration, drug interactions are unlikely.**

**Key words:** $H_1$-receptor antagonist, Levocabastine, Tolerability, Topical antihistamine

### Introduction

Histamine $H_1$-receptor antagonists are widely considered to be a primary treatment option for the clinical management of allergic rhinoconjunctivitis. However, although the clinical efficacy of second generation non-sedating oral antihistamines is well documented,[1] topical therapy may be preferable due to the reduced potential for systemic adverse effects.

Topical therapy available for the treatment of allergic conjunctivitis includes corticosteroids, sodium cromoglycate and vasoconstrictors, either administered alone or in combination with an $H_1$-receptor antagonist. Intranasal corticosteroids provide effective relief from nasal symptoms and are generally well tolerated. However, ocular steroid administration should generally be avoided due to an associated risk of glaucoma, cataracts and opportunistic infections.[2] Sodium cromoglycate is a well tolerated agent for the prophylaxis of allergic rhinoconjunctivitis and is suitable for both intranasal and ocular administration. The therapeutic efficacy of this drug is highly variable and maintenance therapy throughout the period of allergen exposure is essential. Topical vasoconstrictors provide effective relief only for nasal obstructive symptoms and are only suitable for short-term therapy due to the potential for rebound vasodilatation during prolonged use.[3,4]

Until recently, topical antihistamines have not been sufficiently potent to enable single agent therapy, necessitating co-administration of a vasoconstrictor. While reports suggest that such combinations may be more effective than vasoconstrictor monotherapy,[5] they are not suitable for long-term use due to the adverse reactions associated with topical vasoconstrictors.

### Levocabastine

Levocabastine is an extremely potent and highly selective $H_1$-receptor antagonist which has been specifically developed for topical ocular and nasal administration.[6] Levocabastine is the most potent antihistamine available to date expressing antihistaminic activity at doses lower than 0.002 mg/kg, making it some 15 000 times more potent than chlorpheniramine.[7] In addition, animal studies have shown that this agent has a very high specificity for $H_1$-receptors being devoid of anticholinergic, antiserotoninergic or antidopaminergic activity at concentrations

© 1995 Rapid Communications of Oxford Ltd considerably greater than effective antihistaminic doses. This suggests that adverse reactions resulting from a lack of receptor specificity are unlikely. Levocabastine is a potent inhibitor of experimentally induced rhinocon-junctivitis providing effective relief from symptoms within minutes of instillation,[8–13] with comparative clinical trials demonstrating that this agent is at least as effective as oral antihistamines in patients with clinical disease.[14–17]

Pharmacokinetic analysis suggests that the potential for systemic adverse effects is very low. Levocabastine is incompletely absorbed after intranasal and ocular administration with systemic availability typically ranging from 30–60% for the eye drops and 60–80% for the nasal spray.[18] Moreover, as the amount of drug applied topically is small, the plasma concentration of levocabastine attained via these routes of administration is extremely low with $C_{max}$ values in the range of 0.2–0.29 mg/l for levocabastine eye drops and 1.4–2.2 mg/l for the nasal spray. Detailed pharmacokinetic–pharmacodynamic modelling confirms those findings, indicating that the clinical benefits seen with this agent are predominantly due to local antihistaminic effects at the site of application.[19] Coupled with the fact that levocabastine is subject to minimal hepatic metabolism, this suggests that the potential for systemic adverse reactions is minimal.

The present review aims to summarize the data available to date concerning the tolerability and safety of levocabastine eye drops and nasal spray with particular reference to implications for the clinical management of patients with allergic rhinoconjunctivitis.

### Tolerability

The tolerability of levocabastine eye drops and nasal spray has been extensively evaluated in clinical trials. The available data suggests that topical levocabastine is well tolerated with an adverse effect profile comparable with that of sodium cromoglycate or placebo. Analysis of data from a total of 1628 patients treated with levocabastine eye drops reveals that local irritation is the most frequently reported adverse event following ocular administration with an incidence of 14%. Of 1758 patients who have received levocabastine nasal spray, headache (4%), nasal irritation (3%), somnolence (3%) and fatigue (2%) have been reported. The overall incidence of adverse experiences on levocabastine eye drops

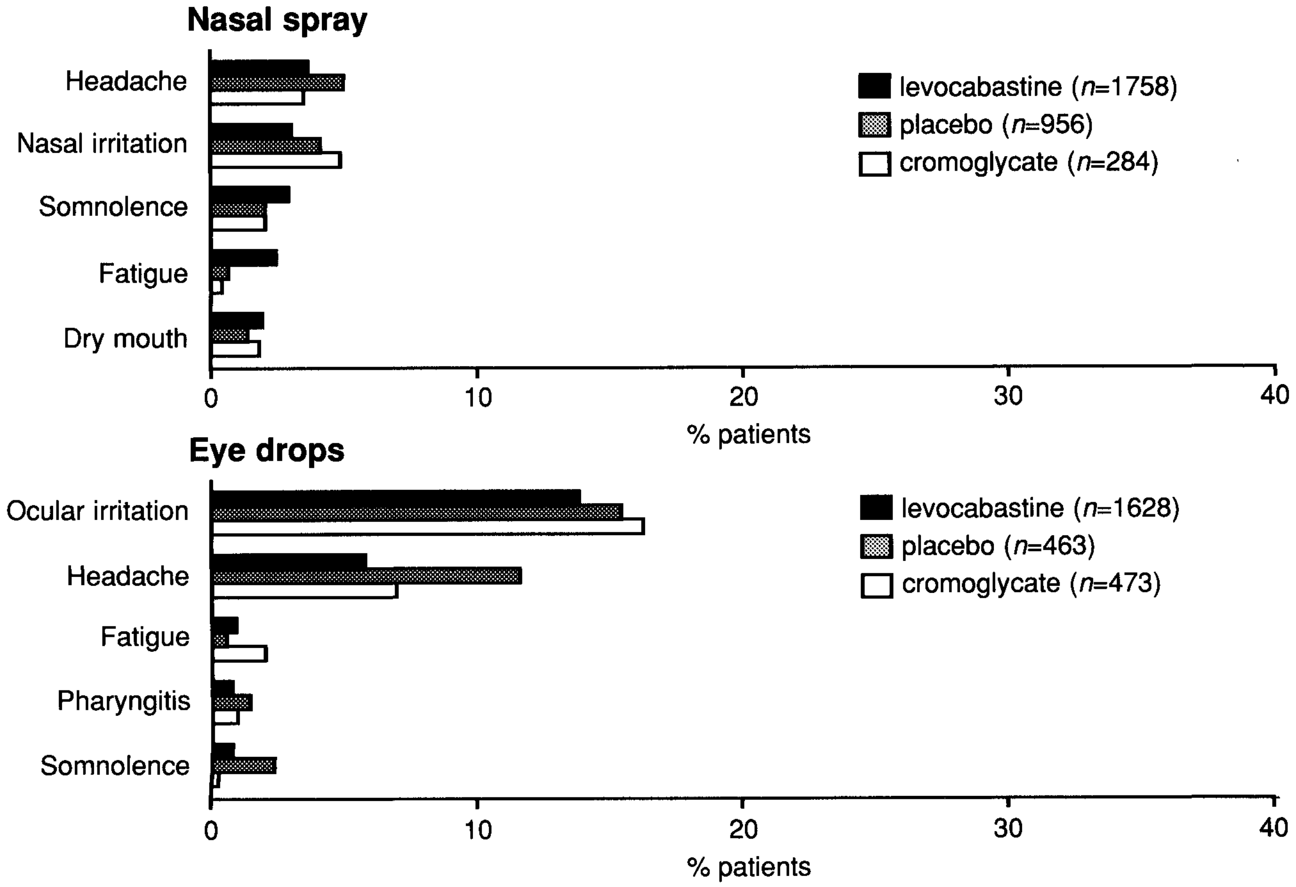

FIG. 1. Incidence of adverse experiences with levocabastine nasal spray and eye drops compared with sodium cromoglycate and placebo.

is 24%. In separate studies the incidence has been comparable for levocabastine and placebo (27 versus 31%), levocabastine and cromoglycate (36 versus 31%) and levocabastine and terfenadine (40 versus 41%). Figure 1 shows the tolerability of levocabastine compared with sodium cromoglycate and placebo.

In addition, no clinically significant changes in biochemical or haematological parameters have been reported during treatment with this topical antihistamine in clinical trials that have performed routine laboratory tests to date. Significantly, the type and frequency of adverse experiences appears to be unrelated to the number of daily applications. Moreover, adverse effects do not appear to be increased by concomitant use of the eye drops and nasal spray compared with use of either formulation alone.

Drug tolerability is a key factor determining choice of therapy in children. Importantly, a study involving 53 children aged between 6 and 15 years reported that levocabastine nasal spray and eye drops are well tolerated in this patient population with type and incidence of adverse reactions similar to those occurring in sodium-cromoglycate-treated children.[20]

*Local tolerance:* It is well documented that intranasal administration of certain drugs and additives, in particular decongestants, can influence ciliary activity of the upper airways.[21] The influence of levocabastine on ciliary beat frequency and mucociliary clearance has been evaluated *in vitro* using human adenoid tissue and *in vivo* following single and multiple dose administration in healthy human volunteers.[22] Levocabastine was seen to have only a small, clinically insignificant effect on ciliary beat frequency in human adenoid tissue. No significant effects on ciliary function were observed following single- and multiple-dose administration in healthy volunteers.

Ocular tolerance of levocabastine has been evaluated extensively. Animal studies have failed to reveal any adverse effects resulting from topical administration of levocabastine eye drops. In an eye irritation study in rabbits, application of levocabastine eye drops four to eight times daily for 4 weeks did not produce ocular changes, and in another study, normal intraocular pressure was found to be maintained in rabbits following application of levocabastine. Levocabastine did not induce dermal sensitization when tested in guinea-pigs using the Magnusson maximization technique.[18]

The local tolerance of levocabastine eye drops has also been extensively evaluated in volunteers and atopic patients during clinical trials using a wide range of objective ophthalmological tests including slit lamp evaluation, tonometry, fluorescein and rose bengal staining, ophthalmoscopy and determination of intraocular pressure, visual acuity and visual fields. These studies have failed to reveal any significant or consistent adverse effects attributable to the topical antihistamine at doses of up to 4 times daily and treatment durations of up to 8 weeks.[23]

As many traditional antihistamines have been associated with anticholinergic activity,[1] which may be expected to influence accommodative capacity, the effects of ocular administration of levocabastine on accommodation have been investigated in healthy volunteers and in patients with glaucoma.[24] Administration of a single dose of levocabastine (0.5 mg/ml) was found to have no effect on accommodative capacity in healthy volunteers. Similarly, accommodative capacity and intraocular pressure were unchanged in patients with glaucoma during twice daily treatment with ocular levocabastine over a period of 2 weeks. These findings are in keeping with the highly selective pharmacological profile of the drug.

*Psychomotor effects of levocabastine:* Many traditional $H_1$-receptor antagonists cross the blood–brain barrier producing unwanted central nervous system effects. Increased daytime somnolence and reduced alertness, are frequent adverse reactions particularly encountered with many first-generation oral antihistamines.[1]

Pharmacokinetic studies indicate that sedative effects would be extremely unlikely in patients receiving intranasal or ocular levocabastine due to the low plasma concentrations of the drug obtained by these routes of administration.[18] Specific studies of psychomotor and cognitive function following topical administration of levocabastine support these findings. Objective assessment of psychomotor performance and subjective assessment of sedation have failed to reveal any central nervous system effects in healthy volunteers treated with levocabastine eye drops (0.5 mg/ml, two drops in each eye, four times daily) over a period of 1 week.[25] Potential central nervous system effects of levocabastine have also been investigated following single- and multiple-dose administration of levocabastine eye drops and nasal spray, and compared with those of oral triprolidine. Performance was assessed following medication by a battery of cognitive and psychomotor tests considered as reliable measures of the sedative effects of psychoactive drugs. In contrast to the significant sedative effects of triprolidine, topical administration of levocabastine eye drops and nasal spray at doses up to 2.0 mg/ml, a dose level four times greater

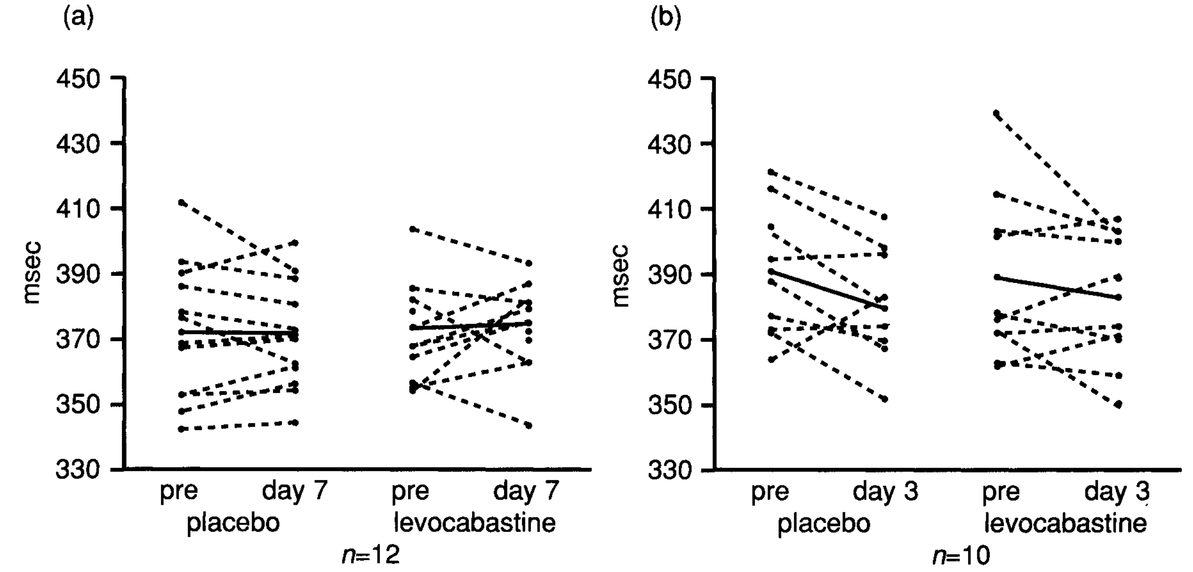

FIG. 2. The effects of levocabastine (a) nasal spray two puffs/nostril and eye drops one drop/eye q.i.d. for 7 days and (b) 3 mg orally for 3 days on the $QT_c$-interval in man.

than normal therapeutic dosages, had no demonstrable effect on central nervous system function in healthy volunteers.[26]

Potential pharmacokinetic and psychomotor interactions between levocabastine nasal spray and alcohol or diazepam have also been investigated in healthy volunteers. No evidence of significant psychomotor interactions between levocabastine and either ethanol or diazepam are apparent.

*Cardiovascular effects of levocabastine:* Possible cardiovascular effects of levocabastine have been assessed *in vitro* on isolated Purkinje fibres of dogs and *in vivo* in animal studies and in human volunteers following oral, ocular and nasal administration. *In vitro* studies on isolated Purkinje fibres revealed no effect of levocabastine on action potential amplitude and action potential duration at 50% and 90% repolarization at doses of up to 2.5 mg/l. Similarly, oral administration of doses of up to 0.16 mg/kg have revealed no significant effect on key cardiovascular parameters in dogs.

More relevantly, levocabastine does not appear to produce significant ECG effects in man. Several studies in healthy volunteers (Fig. 2) have revealed no significant effects on QT- or $QT_c$-intervals after treatment with levocabastine in single or repeated doses, even when the eye drops and the nasal spray are used in combination four times daily (1.2 mg/day) or after administration of oral levocabastine 3 mg daily (for 3 days).[27]

*Drug interactions:* As levocabastine plasma concentrations are extremely low following topical administration and hepatic metabolism is negligible, clinically significant drug interactions are unlikely. But since binding studies have revealed that levocabastine binds to plasma proteins, particularly albumin, the potential for drug interactions involving binding site displacement exists. However, *in vitro* analysis of potential pharmacokinetic drug interactions have identified levocabastine does not alter the plasma protein binding of imipramine, propranolol, diphenylhydantoin, diazepam, cimetidine, indomethacin or ketoconazole although slight increases in the proportion of unbound levocabastine can be identified with drugs which are highly protein-bound such as sulphamethazine (4.5%), tolbutamide (6%) and warfarin (8.1%). Although such changes are minor they should be recognized. Obviously this interaction is of no clinical relevance for levocabastine as its plasma protein binding is only 55%.[18]

### Conclusion

Extensive clinical investigations have shown levocabastine eye drops and nasal spray to be highly effective for the treatment of allergic rhinoconjunctivitis. Levocabastine eye drops and nasal spray are well tolerated with an adverse-effect profile comparable with that of sodium cromoglycate and placebo. Local irritation following administration is the most common adverse reaction reported in levocabastine-treated patients to date with an incidence similar to that seen in placebo- or cromoglycate-treated controls. Importantly, the potential for systemic adverse effects is negligible. As would be expected from its pharmacological profile, levocabastine is devoid of significant systemic activity having no apparent effects on cardiovascular function, psychomotor performance, cognitive function and minimal interaction potential. Coupled with its well documented therapeutic efficacy, these findings indicate that topical levocabastine is an attractive therapeutic option for the treatment of allergic conjunctivitis.

### References

1. Simons FER. $H_1$-receptor antagonists. Clinical pharmacology and therapeutics. *J Allergy Clin Immunol* 1989; **84;** 845–861.
2. Friedlaender MH. Corticosteroid therapy of ocular inflammation. *Int Ophthalmol* 1983; **23:** 175–182.
3. Friedlaender MH. Current concepts in ocular allergy. *Ann Allergy* 1991; **67:** 5–13.
4. Badhwar AK, Druce HM. Allergic rhinitis. *Clin Allergy* 1992; **76:** 789–803.
5. Abelson MB, Paradis A, George MA, *et al.* Effects of Vasocon-A in the allergen challenge model of acute allergic conjunctivitis. *Arch Ophthalmol* 1990; **108:** 520–524.
6. Vanden Bussche G. Levocabastine hydrochloride. *Drugs Future* 1986; **11:** 841–843.
7. Van Wauwe JP. Animal pharmacology of levocabastine: a new type of $H_1$-antihistamine well-suited for topical application. In: Mygind N, Naclerio RM, eds. *Rhinoconjunctivitis: New Perspectives in Topical Treatment of Seasonal Allergic Rhinitis.* Proceedings of the XIIIth International Congress of Allergology and Clinical Immunology. Gottingen: Hogrefe and Huber, 1989; 27–34.
8. Pécoud A, Zuber P, Kolly M. Effect of a new selective $H_1$ receptor antagonist (levocabastine) in a nasal and conjunctival provocation test. *Int Arch Allergy Applied Immunol* 1987; **82:** 541–543.
9. Abelson MB, Smith LM. Levocabastine: evaluation in the histamine and compound 48/80 models of ocular allergy in humans. *Ophthalmology* 1988; **95:** 1494–1497.
10. Palma-Carlos AG, Palma-Carlos ML, Rombaut N. The effect of levocabastine nasal spray in nasal provocation tests. *Int J Clin Pharmacol Res* 1988; **8:** 25–30.
11. Rimås M, Kjellman N-IM, Blychert L-O, Björkstén B. Topical levocabastine protects better than sodium cromoglycate and placebo in conjunctival provocation tests. *Allergy* 1990; **45:** 18–21.
12. Janssens M. Efficacy of levocabastine in conjunctival provocation studies. *Doc Ophthalmol 1992;* **82:** 341–351.
13. Stokes TC, Feinberg G. Rapid onset of levocabastine eye-drops in histamine-induced conjunctivitis. *Clin Exp Allergy* 1993; **23:** 791–794.
14. The Livostin Study Group. A comparison of topical levocabastine and oral terfenadine in the treatment of allergic rhinoconjunctivitis. *Allergy* 1993; **48:** 530–534.
15. Bahmer FA, Ruprecht KW. Safety and efficacy of topical levocabastine compared with oral terfenadine. *Ann Allergy* 1994; **72:** 429–434.
16. Søhoel P, Freng BA, Kramer J, *et al.* Topical levocabastine compared with oral terfenadine for the treatment of seasonal allergic rhinoconjunctivitis. *J Allergy Clin Immunol* 1993; **92:** 73–81.
17. The Swedish GP Allergy Team. Topical levocabastine compared with oral loratadine for the treatment of seasonal allergic rhinoconjunctivitis. *Allergy* 1994; **49:** 611–615.,
18. Dechant KL, Goa KL. Levocabastine. A review of its pharmacological properties and therapeutic potential as a topical antihistamine in allergic rhinitis and conjunctivitis. *Drugs* 1991; **41:** 202–224.
19. Heykants JJP, Snoeck E, Awouters F, Van Peer A. Antihistamines. In: Van Boxel CJ, Holford NHG, Danhof M, eds. *The* In Vivo *Study of Drug Action.* Amsterdam: Elsevier Science, 1992; 337–356.
20. Vermeulen J, Mercer M. A comparison of the efficacy and tolerability of topical levocabastine and sodium cromoglycate in the treatment of seasonal allergic rhinoconjunctivitis in children. *Pediatr Allergy Immunol* 1994; **5:** 209–213.
21. Hermens WAJJ, Merkus FWHN. The influence of drugs on nasal ciliary movement. *Pharmacol Res* 1987; **37:** 294–297.
22. Merkus FWHM, Schüsler-van Hees MTTW. Influence of levocabastine suspension on ciliary beat frequency and mucociliary clearance. *Allergy* 1992; **47:** 230–233.
23. Janssens M, Blockhuys S. Tolerability of levocabastine eye drops. *Doc Ophthalmol* 1993; **84:** 111–118.
24. Wolf S, Wohlrab TM, Remky A. Absence of effects of levocabastine eye-drops on accommodative capacity in volunteers and on intraocular pressure in glaucoma patients. *Eur J Ophthalmol* 1995; in press.
25. Arriaga F, Rombaut N. Absence of central effects with levocabastine eye-drops. *Allergy* 1990; **45:** 552–554.
26. Rombaut N, Bhatti JZ, Curran S, Hindmarch I. Effects of topical administration of levocabastine on psychomotor and cognitive function. *Ann Allergy* 1991; **67:** 75–79.
27. Janssens M. Absence of ECG effects with the topical antihistamine, levocabastine. *J Allergy Clin Immunol* 1995; in press.
